# Does ambient air quality standard contribute to green innovation of enterprises in China? Implications for environmental protection and public health

**DOI:** 10.3389/fpubh.2022.997864

**Published:** 2022-11-10

**Authors:** Zhi-feng Zhang, Hao-dong Xu, Shuang-shuang Shan, Hong-yan Duan, Yu-qi Lu, Yi-pin Lyu

**Affiliations:** ^1^School of Economics, Qingdao University, Qingdao, China; ^2^School of Foreign Language Education, Qingdao University, Qingdao, China; ^3^Department of Economics, The University of Sheffield, Sheffield, United Kingdom; ^4^School of Marxism, East China University of Political Science and Law, Shanghai, China; ^5^College of Engineering and Applied Sciences, The State University of New York at Stony Brook, Stony Brook, NY, United States

**Keywords:** Ambient Air Quality Standard, green innovation, environmental protection, public health, difference-in-difference model, compliance cost effect, innovation offset effect

## Abstract

In the post-COVID-19 era, environmental pollution has been a serious threat to public health. Enterprises are in urgent need of enhancing green technology innovation as the main source of pollutant emissions, and it is necessary for governments to support green innovation of enterprises to reduce pollutant emissions and promote public health. In this context, this paper investigates whether the Ambient Air Quality Standard (AAQS) implemented in 2012 in China contributes to green innovation of enterprises, to provide implications for environmental protection and public health. By using panel data of Chinese A-share listed companies from 2008 to 2020, this study adopts the difference-in-difference model to analyze the policy impact of environmental regulation on green innovation of enterprises and its internal mechanism. The results show that AAQS has significantly improved the green innovation of enterprises. Furthermore, AAQS affects the green innovation of enterprises by virtue of two mechanism paths: compliance cost effect and innovation offset effect. On the one hand, AAQS leads to an increase in production costs of enterprises, thus inhibiting green innovation activities of enterprises. On the other hand, AAQS encourages enterprises to increase R&D investment in green technology, thus enhancing their green innovation. In addition, the impact of AAQS on firms' green innovation has heterogeneous characteristics. Our findings not only enrich the studies of environmental regulation and green innovation of enterprises but also provide policymakers in China and other developing countries with implications for environmental protection and public health improvement.

## Introduction

**Figure 1:**
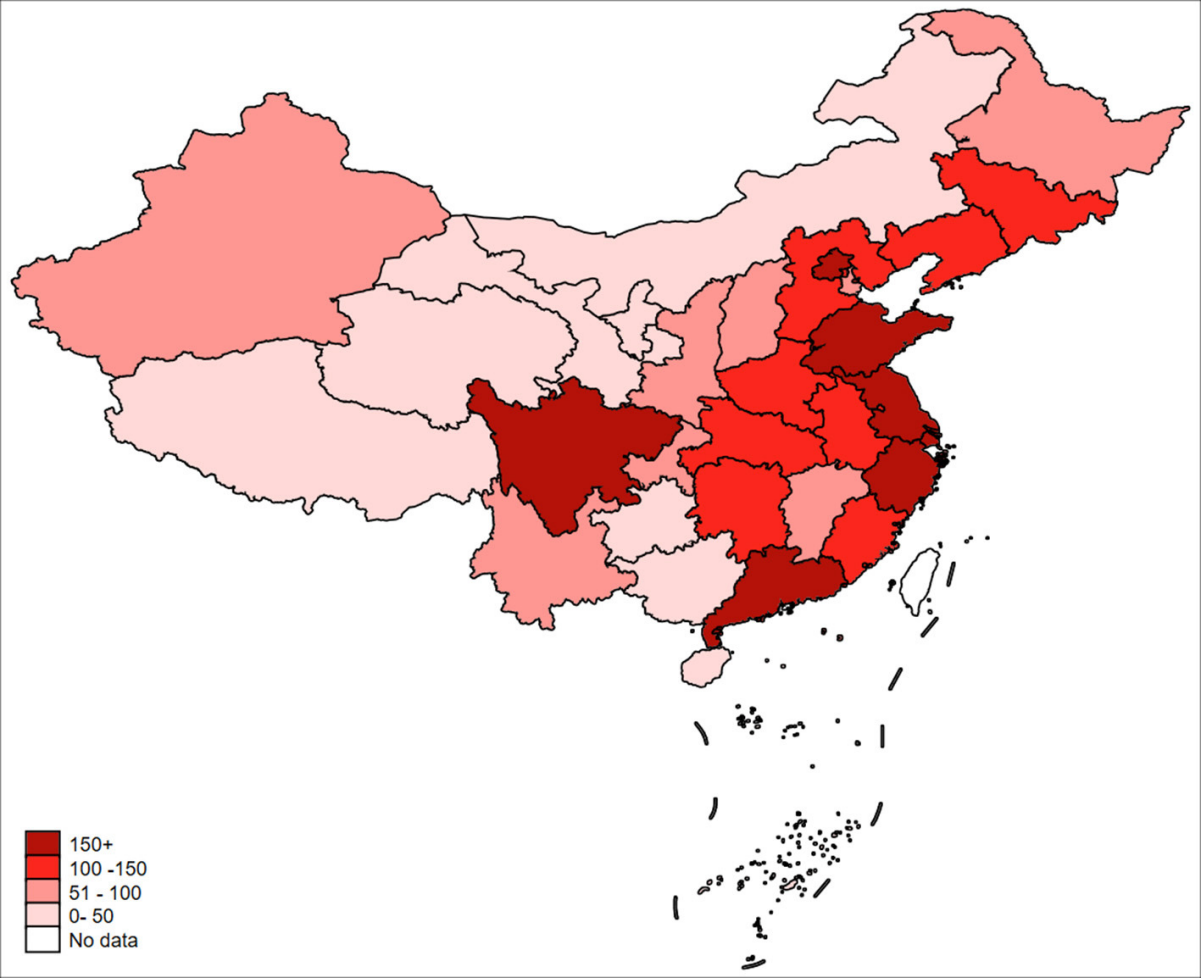
Severity of air pollution in each province in China.

**Figure 2:**
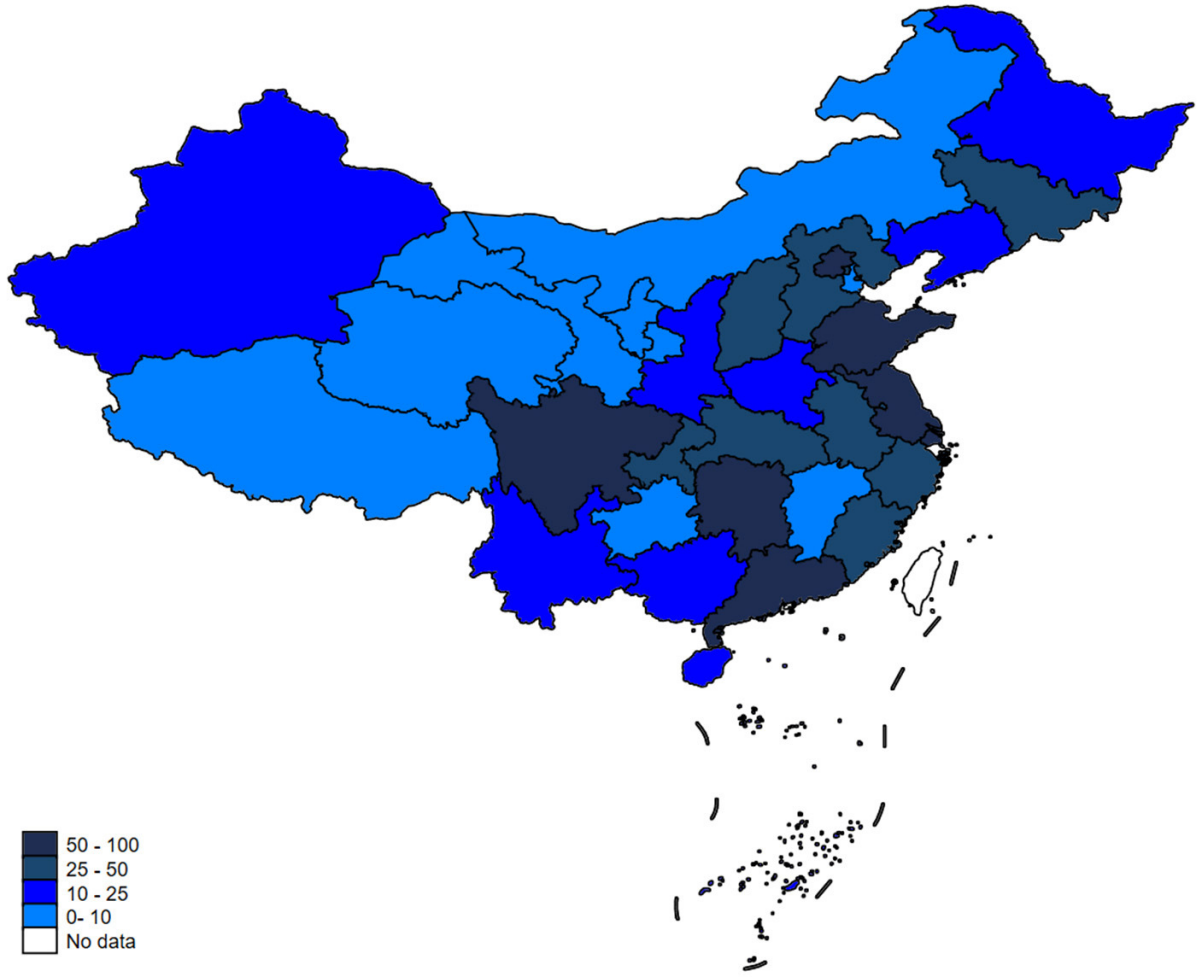
Provincial distribution of the number of national air quality monitoring sites in China.

Environmental quality has been deteriorating over the years due to over-emitted carbon dioxide around the world (1). According to the World Health Organization (WHO), ~7 million people worldwide die from environmental pollution every year (2), which seriously threatens public health. Green development and green transformation have become important issues for many countries (3). China, as the world's largest carbon emitter in the world (4), is facing increasingly serious environmental pollution problems (5). The research shows the number of deaths caused by air pollution in China reached ~1.2 million by 2010 (6, 7). In this context, as the main source of air pollution emissions, enterprises are in urgent need of achieving green development and green transformation (8). Therefore, China's Ministry of Environmental Protection and the State Administration of Quality Supervision and Inspection jointly released the “Notice on Ambient Air Quality Standard” (https://www.mee.gov.cn/gkml/hbb/bgg/201202/) in February 2012 and further announced the “Ambient Air Quality Standard Phase I Monitoring Implementation Plan” (https://www.mee.gov.cn/gkml/hbb/bgt/20120521/) on 21 May 2012. This policy document requires that air quality data from the 74 pilot cities should be released in real time by the national monitoring sites to the public, the government, and the news media starting from 1 January 2013. The list of 74 pilot cities can be found in Supplementary Table S1. The establishment of national monitoring sites depends on the severity of air pollution (9, 10). We display the implementation of the Ambient Air Quality Standard (AAQS) in the following diagrams. As is shown, Figure 1 presents the air quality index of 74 pilot cities in China at the provincial level, and Figure 2 shows the distribution of national air quality monitoring sites in China after the implementation of AAQS. The larger value of the air quality index (AQI) means the more severe the air pollution is. In Figure 1, the lighter to darker colors represent the values of air quality index from small to large. In Figure 2, the lighter to darker colors represent the number of national air quality monitoring sites from less to more.

The implementation of the “Ambient Air Quality Standard Phase I Monitoring Implementation Plan” calls for real-time, interference-free, full coverage of air quality monitoring across China, which increases the opportunity cost of local government environmental governance inaction, facilitates public environmental oversight, and significantly changes the government's original motivation and means of environmental governance (9). Moreover, the implementation of AAQS has stimulated enterprises to invest in energy saving and emission reduction and reduce or shut down high-polluting production. It encourages enterprises to develop green innovation for the achievement of green transformation (11). Hence, these green innovation activities tend to mitigate environmental pollution and promote public health (12). China is still a developing country with low per capita income, and the number of deaths remains relatively high (13, 14). Environmental pollution, ecological environment damage, and other problems seriously threaten the health of Chinese residents (15). Therefore, it is necessary for policymakers to formulate environmental policies that mitigate environmental pollution and improve public health (16). The impact of AAQS on green innovation of enterprises in China is worth testing, and the implications for environmental protection and public health are worth exploring. The novelty of this research and the archival values of the results are as follows.

- The research on AAQS and green innovation enriches the existing studies of the impact of environmental regulation on green innovation of enterprises. Taking the implementation of AAQS as a quasi-natural experiment, this study introduces the compliance cost effect and the innovation offset effect (3) and investigates how the implementation of AAQS influences green innovation by DID model, which can be considered an innovative approach to policy assessment. The combination of theoretical analysis and empirical analysis provides a great research method for the future of scientific literature.
- A series of empirical analyses in detail depicts the impact and intrinsic mechanism of AAQS on green innovation of enterprises, which include heterogeneity analysis, mechanism analysis, PSM-DID, and excluding the impact of other policies. The existing literature proved that AAQS can enhance corporate green innovation. However, the intrinsic mechanism and heterogeneous characteristics of AAQS on green innovation need to be further investigated. Triple-differences model, mediating effect analysis, and PSM-DID estimation provide a new perspective to conduct thorough empirical research.
- The proposed impact of AAQS implementation on green innovation of enterprises *via* the difference-in-difference model is essential to environmental protection and public health. Our findings of the study not only enrich the empirical evidence on environmental policies and firms' green innovation activities but also provide implications for governments to formulate environmental policies that mitigate environmental pollution and improve public health.

The remainder part of this paper is structured as follows: the “Literature Review” section summarizes the relevant studies. In the section “Theoretical Analysis and Research Hypothesis,” the theoretical mechanism of the impact of AAQS on green innovation is described, including the compliance cost effect and innovation offset effect, and the research hypotheses are proposed. The data, variables, and empirical methods utilized in this study are described in the section “Materials and Methods.” Our empirical findings are presented in the “Results” section. The section “Discussion” discusses the results, and finally, the section “Conclusion and Implications” draws the main findings from the empirical analysis, makes relevant theoretical implications and policy recommendations for environmental protection and public health, and presents the limitation and future directions.

## Literature review

Research on the impact of environmental regulation on green innovation has focused on the Porter hypothesis. Several studies supported the Porter hypothesis and proved that environmental regulation can enhance the green innovation level of enterprises (17, 18). Qi et al. (19) concluded that both command-and-control and market-based incentive environmental regulations significantly promote green innovation. It is generally accepted that command-and-control environmental regulation mainly includes emission reduction schemes, energy regulation, government subsidies, and environmental enforcement and supervision, while market-based incentive environmental regulation mainly comprises carbon emissions trading, environmental rights trading, and so on (20–22). In recent years, Yi et al. (23) classified environmental policy instruments into command-control, market-incentive, and social-will types. The impact of different types of environmental policy instruments on green technology innovation is further examined (24). Xie et al. (25) examined the positive effects of command-and-control and market-based incentive environmental policies on green productivity. However, some studies disagreed with the Porter hypothesis. Dean et al. (26) pointed out that in order to meet the environmental standard, companies purchase appropriate pollutant-controlled equipment or reduce pollution from the end, which leads to a significant increase in production costs and a corresponding reduction in R&D investment. Shi and Xu (27) also concluded that environmental policies reduced firms' incentives to engage in green innovation activities and investment. Jing and Zhang (28) showed that strict environmental regulations would significantly increase green total factor productivity based on manufacturing industry segments, which is contrary to the Porter hypothesis. In addition, some studies have shown that the relationship between environmental regulation and green innovation has an inverted U-shaped curve (29, 30).

In terms of indicators for measuring green innovation level of enterprises, some scholars have used green patent data to measure the green innovation (8, 19). Deng et al. (31) used three metrics including green patent, green invention patent, and green utility model patent to measure firms' green innovation capabilities. In addition, some studies have used green total factor productivity as a proxy for green innovation and examined the green innovation effect of environmental regulation accordingly (32).

The implementation of AAQS provides a new perspective, as opposed to existing environmental regulatory policies such as emission reduction programs, energy controls, government subsidies, and environmental inspections and enforcement (33, 34). Greenstone et al. (35) conducted the quasi-natural experiment regarding phased implementation of new air quality standard in pilot cities and found that the introduction of pollutant-monitoring automation technology and real-time reporting of pollution data could effectively mitigate the environmental information asymmetry between the central and local governments. Zhang et al. (9) conducted a quasi-natural experiment with 74 pilot cities implementing the new air quality standard and found that AAQS led to increased environmental requirements by local officials, thus influencing corporate environmental decisions.

This paper makes several contributions: first, the existing literature focuses on the research perspectives of environmental equity trading markets, low-carbon cities plan, environmental target responsibility systems, emission charges, and environmental subsidies (36–38). Instead, this paper provides theoretical and empirical evidence that environmental regulation affects green innovation of enterprises based on the implementation of AAQS. We creatively analyze the policy effect of AAQS to study the impact of environmental regulation on green innovation of enterprises by employing difference-in-difference (DID) model. Second, this paper uses three measurements of the green innovation of enterprises in the empirical analysis, rather than single indicators used in the previous research. We test five types of heterogeneity of the policy effect of AAQS by employing triple-differences model. Besides, we analyze the impact mechanism of AAQS on green innovation to confirm compliance cost effect and innovation offset effect by virtue of mediating effect methods, which are illustrated in detail in the theoretical analysis, and we propose four research hypotheses accordingly. Third, the findings of the study not only enrich the empirical evidence on environmental policies and firms' green innovation activities but also provide implications for governments to formulate environmental policies that mitigate environmental pollution and improve public health.

## Theoretical analysis and research hypothesis

Based on the above literature review, this section demonstrates the internal mechanism of the impact of environmental regulation on green innovation of enterprises from the theoretical perspective, which provides the theoretical basis for the mechanism analysis. Environmental regulation has two impact mechanisms affecting green innovation: the compliance cost effect and the innovation offset effect (3). Then, we put forward several research hypotheses according to the theoretical mechanism analysis according to the following literature.

AAQS serves as a key tool to enhance the transparency of air quality information and achieve multi-subject integration in ecological protection and high-quality development (10). AAQS greatly increases the opportunity cost of local government environmental governance inaction, facilitates public environmental oversight, and raises the probability of punishment for corporate pollution emissions. It suggests that the implementation of AAQS significantly changes the government's original motivation and means of environmental governance (9). Moreover, the implementation of AAQS has stimulated enterprises to invest in energy saving and emission reduction and reduce or shut down high-polluting production. It encourages enterprises to develop green innovation for the achievement of green transformation (11). Based on the above discussion, we propose the first hypothesis in our study:

**Hypothesis 1 (**H1**)**. AAQS has a positive impact on green innovation of enterprises.

From the perspective of static analysis, the compliance cost effect assumes that the level of technology, resource allocation, and firm demand have reached a steady state before the implementation of environmental regulations and that firms have already made the optimal choice between costs and benefits (2). Strict environmental regulations inevitably lead to higher costs of treating and controlling pollution, and this part of the cost can crowd out business resources (39). In order to reduce pollutant emissions to within national Ambient Air Quality Standard, companies need to make capital investments in the selection of production site and the availability of green raw materials. In terms of management, companies need to train professionals in pollution control, which raises management costs (3). In addition, strict environmental regulations make the external environment uncertain for enterprises. Therefore, enterprises need to invest more money to obtain knowledge and information about green technology innovation. This series of environmental expenditures will inevitably increase the financial pressure on enterprises, crowding out the resources invested in green technology innovation, thus hindering enterprises to carry out green technology innovation (31). Based on the above theoretical analysis, we propose the second hypothesis in the study:

**Hypothesis 2 (**H2**)**. AAQS increases firms' production costs and thus inhibits green innovation of enterprises.

The innovation offset effect indicates that firms will change their situation accordingly in a dynamic economic framework. In other words, environmental regulations make firms change their business and innovation models to respond to environmental regulations and reduce policy costs by improving their technological innovation (40). As environmental enforcement continues to strengthen, companies have two choices facing strict environmental regulations: either technological innovation or pollution control. Corporate R&D investment is the main source of technological innovation, and it is believed that increasing corporate R&D investment will promote higher levels of corporate technological innovation (41). When the environmental regulation factor is added, the technology level can be improved if the environmental regulation increases the R&D investment (42). Based on the principle of profit maximization, enterprises will increase R&D investment, improve resource allocation and production management efficiency, and reduce policy costs, thus enhancing corporate competitiveness with the effect of reasonable environmental regulations (31, 40). Based on the above theoretical analysis, we propose the third hypothesis in the study:

**Hypothesis 3 (**H3**)**. AAQS increases R&D investment of enterprises, thus enhancing the green innovation.

The compliance cost effect emphasizes that environmental regulations lead to higher costs for firms to treat pollution, thus limiting them to a certain extent to progress green innovation (43). The innovation compensation effect argues that with the guidance of environmental regulation policies, enterprises increase their R&D investment in green production technologies. Hence, what is the total effect when compliance cost effect and innovation compensation effect act together? The effect of increasing R&D investment of clean production companies could be more obvious (44, 45), prompting heavy-polluting enterprises to promote green technology upgradation (46). Based on the above discussion, we propose the fourth hypothesis in our study:

**Hypothesis 4 (**H4**)**. The innovation offset effect of AAQS is greater than the compliance cost effect, leading to a positive total effect.

**Figure 3:**
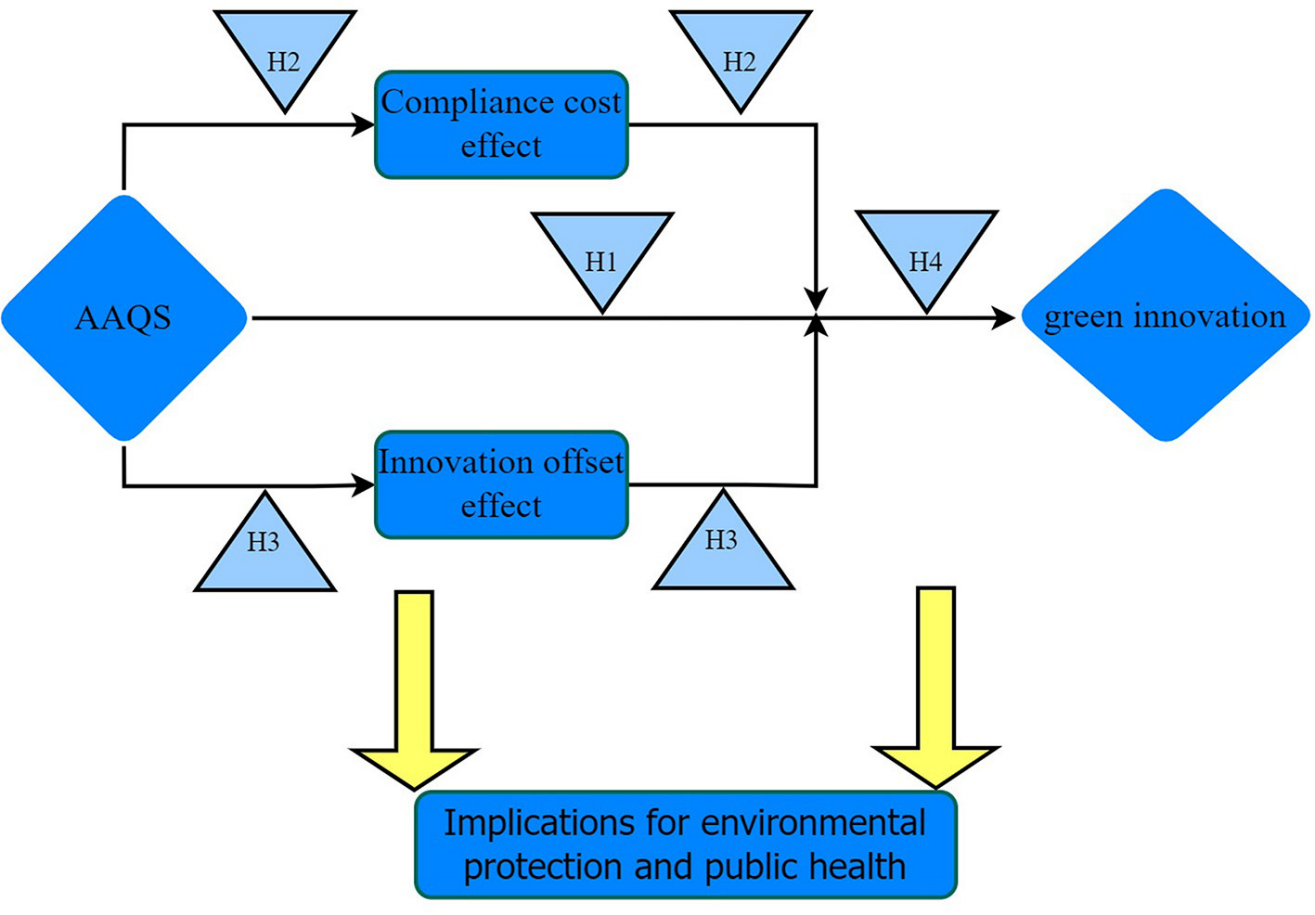
Theoretical framework diagram.

Based on the above theoretical analysis and proposed research hypotheses, this paper establishes theoretical framework diagram of our study, as shown in Figure 3.

## Materials and methods

### Data and variables

This study selects patent data and corporate characteristics data of A-share listed companies in China during the period from 2008 to 2020 for the empirical study. The previous studies generally select the number of green patent applications as the measure of green innovation (8, 47). In order to reflect the differences in the degree of innovation and value of green patent applications, this paper distinguishes green patent applications into green invention patent applications and green utility model patent applications. Considering that invention patents best reflect a company's original innovation activities (48), we use the number of green invention patent applications to portray the quality of a company's green innovation. Meanwhile, the number of green utility model patent applications is used as a comparative indicator. Therefore, this paper selects the number of green patent applications, the number of green invention patent applications, and the number of green utility model patent applications as the measurement of green innovation (10, 49). We obtained the international patent classification numbers of invention patent applications and utility model patent applications of A-share listed companies from the China Research Data Service (CNRDS) (https://www.cnrds.com/) and matched them with the “IPC Green Inventory” (https://www.wipo.int/classifications/ipc/en/) issued on 16 September 2010, by the World Intellectual Property Organization (WIPO). If the classification number of a patent application is within the “IPC Green Inventory”, the patent application is considered to be a green patent application. Otherwise, it is considered to be a non-green patent application.

This identification method is consistent with those used by the studies of Li and Xiao (17), Wurlod and Noailly (50), and Fang and Na (51). To eliminate the problem of right-skewed distribution of patent applications data, this study adds 1 to the above three metrics and takes the natural logarithm, consequently obtaining Patent, Inva, and Uma, respectively (52, 53). In the empirical analysis, we use Patent, Inva, and Uma as explained variables to test the effect of environmental regulations on green innovation of enterprises. As for control variables, this paper selects equity concentration (Top), cash ratio (Cash), debt financing cost (Debt), leverage ratio (Lar), return on equity (ROE), firms' total asset (Size), Tobin's Q (Tobin), capital intensity (Capital), and stock annual return (Ret) (8, 19, 47, 54). Control variables and other data were obtained from the CSMAR database (https://www.cndata1.csmar.com/). The variables in our study are represented in Table 1. To eliminate the effect of extreme values, all variables are winsorized by 1%. The descriptive statistics of each variable are shown in Table 2.

**Table 1:** Variable definitions.

**Variable Type**	**Variables**	**Statistic**	**Variable Definition**
Explained variable		Patent	Ln (the number of green patent applications+1)
	Green innovation	Inva	Ln (the number of green invention patent applications +1)
		Uma	Ln (the number of green utility model patent applications +1)
Explanatory variables	Policy dummy variable	D	D is the interaction term between treat and post, which is policy dummy variable in the DID model
Control variables	Equity concentration	Top	Shareholding ratio of the largest shareholder
	Cash ratio	Cash	Ending balance of cash and cash equivalents/current liability
	Debt financing cost	Debt	Finance costs/interest-bearing liabilities
	Leverage ratio	Lar	Ending balance of total liability/ending balance of total asset
	Return on equity	ROE	The ratio of net income to total average equity
	Firms' total asset	Size	Total enterprise assets ending balance
	Tobin's Q	Tobin	Market value of tradable shares+par value of non-tradable shares/total asset-net intangible asset-net goodwill
	Capital intensity	Capital	The ratio of total asset to sales revenue
	Stock annual return	Ret	Annual return of individual stocks

**Table 2:** Descriptive statistics.

**Statistic**	**Unit**	**Observations**	**Means**	**Standard**	**Min**	**Max**
				**deviation**		
Patent	–	20,254	1.17067	1.60265	0	6.13988
Inva	–	20,254	0.33709	0.78515	0	3.89182
Uma	–	20,254	1.11628	1.56107	0	6.02345
D	–	20,254	0.23976	0.42695	0	1
Top	%	20,216	34.4238	15.2675	8.4	74.98
Cash	%	20,237	0.55422	0.83127	0	5.52406
Lar	–	20,238	0.56286	0.26216	0.77752	1.68648
Debt	–	17,757	0.07662	0.17149	−0.0378	0.89255
ROE	–	20,238	0.04139	0.20634	−1.3407	0.41322
Size	billion CNY	20,238	31	133	0.153	1170
Tobin	–	18,132	2.13083	1.77958	0.86135	12.6265
Capital	–	20,254	2.89467	3.99880	0.34834	29.8027
Ret	%	17,099	0.39764	1.77303	−0.7865	14.9708

### Empirical model

Taking the Ambient Air Quality Standard implemented in China in 2012 as a quasi-natural experiment, this paper uses DID model to analyze the policy effect of environmental regulation on green innovation of enterprises and its impact mechanism. This paper adopts the difference-in-difference (DID) model, difference-in-difference estimation after propensity score matching (PSM-DID), and the triple-differences model in the empirical analysis, according to the studies of (8, 10, 31). The specific DID model is set as shown in Equation (1).


(1) $$\begin{array}{l} \text{Innovatio}n_{it}\;=\;\alpha \;+\;\beta_{1}D_{it}\;+\;\beta_{2}\text{contro}l_{it}\;+\;{\mu_{i}+\lambda }_{t}\;+\;\varepsilon_{it} \end{array}$$

In Equation (1), *Innovation*_*it*_ denotes the green innovation of enterprises, measured by three metrics including *Patent*_*it*_, *Inva*_*it*_, and *Uma*_*it*_. *D*_*it*_ represents the policy dummy variable, which is the interaction item between *Treat*_*i*_ and *Post*_*t*_. *Treat*_*i*_ is a dummy variable for the policy pilot cities. The implementation of “Ambient Air Quality Standard Phase I Monitoring Implementation Plan” provides 74 pilot cities for the study. Listed companies in 74 pilot cities are used as the experimental group, and the remaining are taken as the control group. The experimental group takes the value of *Treat*_*i*_= 1 while the control group takes the value of *Treat*_*i*_= 0. *Post*_*t*_ is a dummy variable for the policy pilot year. AAQS was officially proposed in 2012. Therefore, the value of *Post*_*t*_ is taken as 1 in 2012 and later, otherwise the value of *Post*_*t*_ is 0. β_1_ is the core indicator to measure the policy effect of AAQS. If β_1_ is significantly positive, it indicates that the implementation of AAQS could promote green innovation and green development of enterprises. α represents the constant term. *Control*_*it*_ represents the control variable. λ_*t*_ is the year-fixed effect, μ_*i*_ is the firm-fixed effect, and ε_*it*_ denotes the random error term.

## Results

### Benchmark regression analysis

This study selects Patent, Inva, and Uma as explained variables to conduct regression by using Equation (1), respectively. The results of the benchmark regression analysis are presented in Table 3. Columns (a), (c), and (e) do not include control variables, while control variables are added in columns (b), (d), and (f) based on columns (a), (c), and (e), respectively. The results show that the coefficient of *D*_*it*_ is significantly positive regardless of whether Patent, Inva, or Uma is used to measure green innovation of enterprises. β_1_ is significant at the 1% level when no control variables are included, whereas significant at the 5% level when control variables are included. The number of green patent applications, green invention patent applications, and green utility model patent applications has increased significantly, with an increase of 0.165, 0.081, and 0.173, respectively. It is suggested that the implementation of AAQS has a significant positive effect on green innovation of enterprises, which confirms hypothesis 1. The positive effect of AAQS on green innovation of enterprises may be derived from the well resolution of the problem of information asymmetry on the extent of air pollution. After the implementation of AAQS, environmental enforcement represented by local governments and National Environmental Protection Administration increases rapidly. There is more public participation in environmental protection, by means of complaints and reports such as phone calls and letters. Meanwhile, media coverage of environmental pollution is likely to be more active. In the process of environmental enforcement, public supervision, and media oversight, companies will accelerate green innovation activities that significantly reduce pollution externalities, especially high-quality green innovation (10).

**Table 3:** Results of benchmark regression.

**Variables**	**Patent**		**Inva**		**Uma**	
	**(a)**	**(b)**	**(c)**	**(d)**	**(e)**	**(f)**
D	0.148***	0.165**	0.067***	0.081**	0.155***	0.173**
	(0.044)	(0.069)	(0.024)	(0.038)	(0.043)	(0.067)
Top		−0.053		−0.429***		−0.442***
		(0.036)		(0.040)		(0.065)
Cash		0.032***		0.019*		0.022
		(0.017)		(0.011)		(0.035)
Lar		−0.070***		−0.042		−0.013
		(0.020)		(0.059)		(0.096)
Debt		−0.229		−0.077		−0.189
		(0.139)		(0.068)		(0.131)
ROE		0.023		0.020		0.027
		(0.061)		(0.031)		(0.040)
Size		0.355***		0.317***		0.324***
		(0.054)		(0.027)		(0.051)
Tobin		0.036*		0.014		0.034*
		(0.011)		(0.005)		(0.015)
Capital		0.368		0.300		0.331
		(0.037)		(0.035)		(0.037)
Ret		0.402***		0.433***		0.469***
		(0.060)		(0.035)		(0.058)
Constant	0.879***	1.164***	0.181***	0.260***	0.843***	1.114***
	(0.022)	(0.109)	(0.012)	(0.067)	(0.021)	(0.102)
Firm-fixed effect	Control	Control	Control	Control	Control	Control
Year-fixed effect	Control	Control	Control	Control	Control	Control
Observations	20,254	17,099	20,254	17,099	20,254	17,099
R-squared	0.720	0.734	0.701	0.754	0.738	0.745

The parentheses indicate the clustered standard errors at the prefecture-level firm level. ***, **, and * indicate significance at the 1, 5, and 10% levels, respectively.

### Robustness test

In this study, five methods are used to test the robustness of the regression results (3, 13, 31). The first method is the parallel trend test. The second method is the placebo test. The third is the replacement of the explained variables. The fourth method is PSM-DID. The fifth method is the exclusion of the influence of other policies.

#### Parallel trend test

**Figure 4:**
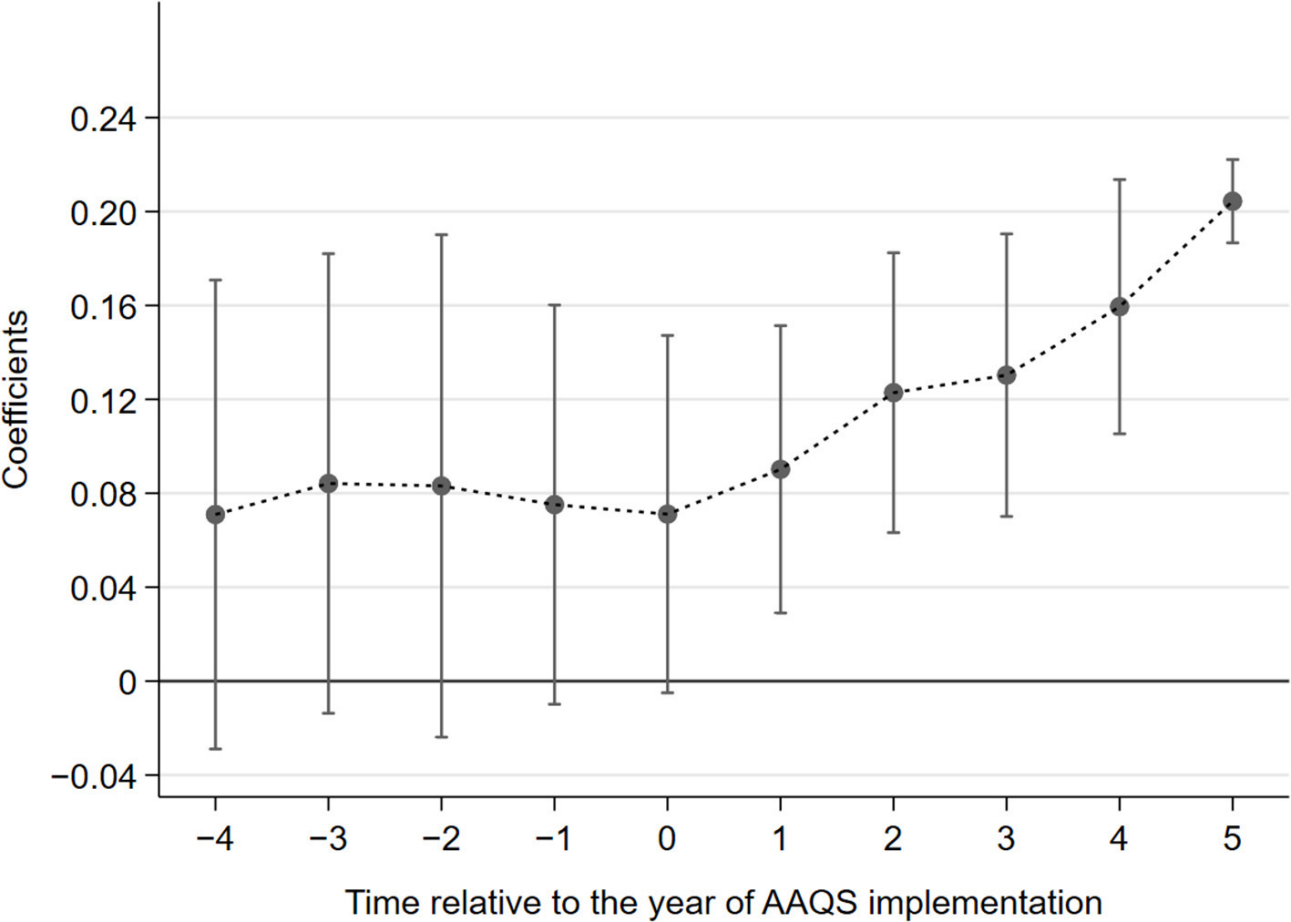
Parallel trend test.

Conforming to the parallel trend assumption is an important prerequisite for using the DID model. The parallel trend assumption means that the experimental group and the control group should have the same trend of change before the policy is implemented. Referring to the test methods of parallel trend assumption in related literature (13, 55), the following model is constructed for parallel trend test, as shown in Equation (2).


(2) $$\begin{array}{l} \text{Innovatio}n_{it}\;=\;\beta_{0}\;+\;\beta_{i}\sum_{k\;=\;-\;4}^{5}{D^{k}}_{it}\;+\;\tau \text{contro}l_{it}\;+\;\mu_{i}\\ \;+\;\lambda_{t}+\varepsilon_{it} \end{array}$$

where the value of ${D^{k}}_{it}$ is based on the year of AAQS implementation, and the value of k denotes the time relative to the year of AAQS implementation. In 2012, *k* takes the value of 0. ${D^{k}}_{it}$ takes the value of 0 in the *k* years before 2012, and ${D^{k}}_{it}$ takes the value of 1 in the *k* years after 2012. Figure 4 shows the graphical results of the parallel trend test. The horizontal axis indicates the time relative to the year of AAQS implementation (k), ranging from −4 to 5. The vertical axis denotes the estimated coefficient of ${D^{k}}_{it}$. The parallel trend test shows that before the implementation of AAQS, there is no significant trend difference in the level of green innovation between the listed companies in the experimental group and the control group. However, after the implementation of AAQS, the number of green patent applications began to increase significantly, which indicates that AAQS has a significant effect on the green innovation of enterprises. Therefore, DID model in this study satisfies the parallel trend assumption.

#### Placebo test

**Figure 5:**
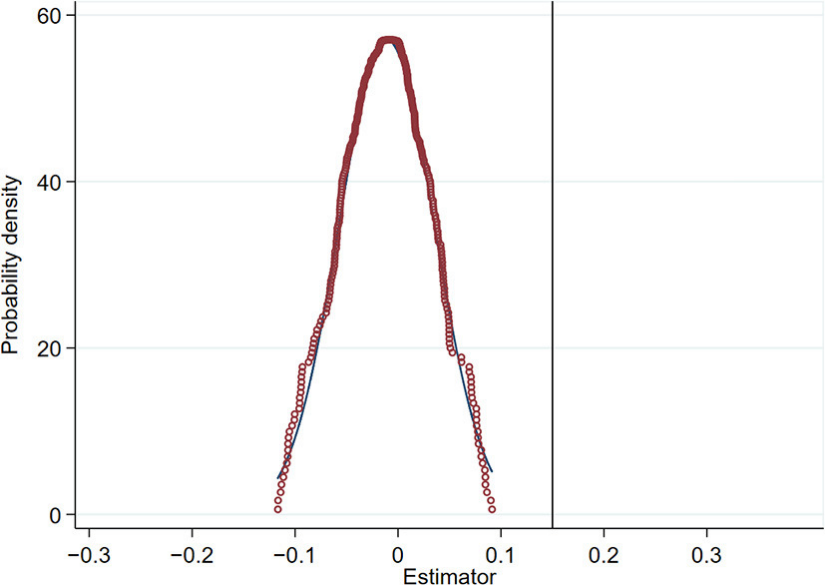
Placebo test.

The benchmark regression analysis demonstrates that the implementation of AAQS plays a significant role in promoting green innovation of enterprises. In order to avoid that the policy effect of AAQS stems from other unobservable factors, this paper further conducts the placebo test. The core idea of placebo test is to estimate a fictitious treatment group or a fictitious policy time. All listed companies in the pilot cities in the experimental and control groups in the previous section are randomly sampled again after disordering them. Then, some companies are randomly selected as the fictitious experimental group and assigned the value of *D*_*it*_ = 1, while other companies are assigned the value of *D*_*it*_ = 0. We re-estimate Equation (1) based on the policy dummy variables constructed by re-random sampling to obtain the estimation results. This process is repeated 1,000 times, and the estimated coefficients of *D*_*it*_ in fictitious treatment group can be obtained to plot the probability density curve (13). Figure 5 shows that the probability density plot shows normal distribution characteristics, and the coefficient estimates of *D*_*it*_ are significantly different from the mean value of the kernel density distribution. It indicates that the policy effect of AAQS on firms' green innovation does not originate from other unobservable factors, but stems from the AAQS implementation itself. Therefore, the DID model passes the placebo test.

#### Substitution of the explained variables

Considering that relative indicators of green patents may be more effective than absolute indicators in eliminating unobservable factors outside a certain policy (19), this paper adopts the following relative indicators for robustness test: the proportion of green patent applications to all patent applications in a year (Rpatent), the proportion of green invention patent applications to all invention patent applications in a year (Rinva), the proportion of green utility model patent applications to all utility model patent applications in a year (Ruma) (47). The descriptive statistics of the surrogate variables are shown in Table 4. This section regresses Equation (1) by replacing the explained variables as follows: the number of green patent applications (Patent) is replaced with the proportion of green patent applications to all patent applications (Rpatent). The number of green invention patent applications (Inva) is replaced with the proportion of green invention patent applications to all invention patent applications (Rinva). The number of green utility model patent applications (Uma) is replaced with the proportion of green utility model patent applications to all utility model patent applications (Ruma). Table 5 shows the results of replacing the explained variables.

**Table 4:** Descriptive statistics of surrogate variables.

**Statistic**	**Unit**	**Observations**	**Means**	**Standard deviation**	**Min**	**Max**
				**deviation**		
Rpatent	–	20,254	0.10812	0.20397	0	0.645
Rinva	–	20,254	0.88979	0.20598	0	0.321
Ruma	–	20,254	0.05511	0.08587	0	0.405

**Table 5:** Robustness test results of replacing the explained variables.

**Variables**	**Rpatent**		**Rinva**		**Ruma**	
	**(a)**	**(b)**	**(c)**	**(d)**	**(e)**	**(f)**
D	0.030***	0.024**	0.017**	0.010**	0.013***	0.016***
	(0.010)	(0.008)	(0.013)	(0.011)	(0.002)	(0.003)
Top		0.043		0.028**		0.048
		(0.010)		(0.014)		(0.010)
Cash		0.010***		0.013*		0.011***
		(0.002)		(0.003)		(0.004)
Lar		−0.030		−0.033		−0.085***
		(0.021)		(0.028)		(0.010)
Debt		−0.098		−0.092		−0.021
		(0.091)		(0.090)		(0.026)
ROE		0.014		0.015		0.008
		(0.023)		(0.023)		(0.005)
Size		0.031***		0.017		0.036***
		(0.010)		(0.014)		(0.010)
Tobin		0.027***		0.011***		0.021
		(0.009)		(0.002)		(0.013)
Capital		0.025*		0.008**		0.017***
		(0.013)		(0.003)		(0.004)
Ret		0.032***		0.055***		0.021***
		(0.009)		(0.011)		(0.008)
Constant	0.091***	0.137***	0.909***	0.869***	0.053***	0.055***
	(0.006)	(0.023)	(0.006)	(0.022)	(0.002)	(0.011)
Firm-fixed effect	Control	Control	Control	Control	Control	Control
Year-fixed effect	Control	Control	Control	Control	Control	Control
Observations	20,254	17,099	20,254	17,099	20,254	17,099
R-squared	0.608	0.614	0.609	0.615	0.516	0.588

The parentheses indicate the clustered standard errors at the prefecture-level firm level. ***, **, and * indicate significance at the 1, 5, and 10% levels, respectively.

As shown in Table 5, the coefficients of *D*_*it*_ are 0.030 and 0.024 in columns (a) and (b), respectively. β_1_ is significant at the 1% level when no control variables are added, while significant at the 5% level when control variables are added. In columns (c) and (d), the coefficients of *D*_*it*_ are 0.017 and 0.010, respectively, and both are significant at the 5% level. In columns (e) and (f), β_1_ are 0.013 and 0.016, respectively, and both are significant at the 1% level. The results indicate that the implementation of AAQS plays a significant role in promoting corporate green innovation. It is consistent with the results in Table 3, indicating that the regression findings are accurate.

#### PSM-DID estimation

This study further uses the difference-in-difference estimation after propensity score matching (PSM-DID) for robustness test (11, 49). There are three types of matching methods used in this section, including kernel matching, caliper matching, and nearest neighbor matching (56). The specific steps are as follows. First, the control variables in the benchmark regression model are used as matching feature variables to construct the logit model. Second, the predicted values of the logit model are used as the matching scores to exclude the unsuccessful matched samples. Third, we use the DID estimation for regression, based on the data obtained after kernel matching, caliper matching, and nearest neighbor matching. Table 6 shows the results of PSM-DID estimation. Columns (a), (d), and (g) are the results of PSM-DID when we select Patent as the explained variable. Columns (b), (e), and (h) are the results of PSM-DID with Inva as the dependent variable. Columns (c), (f), and (i) are the results of PSM-DID using Uma as the measure indicator of green innovation. All regressions include control variables. Columns (a)–(c) show the estimation results of kernel matching. Columns (d)–(f) present the results of caliper matching. The estimation results of nearest neighbor matching are shown in columns (g)–(i). It can be seen that the coefficients of *D*_*it*_ are positive and pass the significance test, whether we use kernel matching, caliper matching, or nearest neighbor matching for PSM-DID regression. It indicates that the implementation of AAQS has a significant positive effect on green innovation of enterprises, which is consistent with the results of the benchmark regression analysis. Therefore, the regression results are robust in the study.

**Table 6:** Robustness test results of PSM-DID estimation.

**Variables**	**Kernel matching**			**Caliper matching**			**Nearest neighbor matching**		
	**(a)**	**(b)**	**(c)**	**(d)**	**(e)**	**(f)**	**(g)**	**(h)**	**(i)**
D	0.231***	0.161**	0.106***	0.090**	0.204***	0.152**	0.523***	0.209***	0.496***
	(0.145)	(0.120)	(0.031)	(0.008)	(0.047)	(0.088)	(0.011)	(0.026)	(0.077)
Control variables	Control	Control	Control	Control	Control	Control	Control	Control	Control
Constant	1.176***	0.299***	1.124***	1.196***	1.195***	0.262***	0.260***	1.160***	1.159***
	(0.024)	(0.013)	(0.024)	(0.037)	(0.020)	(0.019)	(0.037)	(0.126)	(0.038)
Firm-fixed effect	Control	Control	Control	Control	Control	Control	Control	Control	Control
Year-fixed effect	Control	Control	Control	Control	Control	Control	Control	Control	Control
Observations	17,099	17,099	17,099	17,099	17,099	17,099	17,099	17,099	17,099
R-squared	0.618	0.613	0.637	0.558	0.577	0.596	0.757	0.724	0.726

The parentheses indicate the clustered standard errors at the prefecture-level firm level. ***, **, and * indicate significance at the 1, 5, and 10% levels, respectively.

#### Exclusion of the influence of other policies

In the benchmark regression, we have concluded that the implementation of AAQS has a significant positive effect on green innovation of enterprises, which confirms hypothesis 1. However, one problem is how to be sure that Ambient Air Quality Standard (AAQS) is the only one responsible for the increase of corporate green innovation? To address this problem, this section attempts to exclude the impact of some other policies except AAQS that affect green innovation of enterprises to verify the conclusions of benchmark regression analysis. Given that some other policies occurred during the sample period, the following two policies may have a certain impact on our results: the first is the green credit guideline promulgated in 2012; the second is the implementation of the green finance reform and innovation pilot zone established in 2017, and we have excluded the impact of the two policies on green innovation. The results of excluding the impact of other policies are shown in Table 7.

**Table 7:** Results of excluding the impact of other policies.

**Excluded policies**	**Patent**		**Inva**		**Uma**	
	**(a)**	**(b)**	**(c)**	**(d)**	**(e)**	**(f)**
Green credit	0.155***	0.149***	0.066***	0.068**	0.164***	0.158***
	(0.044)	(0.048)	(0.024)	(0.027)	(0.043)	(0.047)
Green finance reform and innovation pilot zone	0.120*** (0.020)	0.138*** (0.029)	0.053*** (0.011)	0.054*** (0.016)	0.110*** (0.019)	0.130*** (0.028)
Constant	1.070***	1.161***	0.280***	0.296***	1.022***	1.105***
	(0.055)	(0.076)	(0.030)	(0.045)	(0.052)	(0.073)
Firm-fixed effect	Control	Control	Control	Control	Control	Control
Year-fixed effect	Control	Control	Control	Control	Control	Control
Observations	18,696	15,783	18,696	15,783	18,696	15,783
R-squared	0.742	0.746	0.743	0.752	0.741	0.742

The parentheses indicate the clustered standard errors at the prefecture-level firm level. ***, **, and * indicate significance at the 1, 5, and 10% levels, respectively.

To exclude the possible impact of the above two policies, we conduct the following tests. First, because the green credit guideline was promulgated in 2012, we remove all data in 2012 and then re-estimate Equation (1). The results are shown in the first row in Table 7. Similarly, Equation (1) was estimated by excluding all data for 2017 to exclude the impact of the green finance reform and innovation pilot zone established in 2017. The results are shown in the second row in Table 7. The control variables are not added in columns (a), (c), and (e). We added variables in columns (b), (d), and (f). The results show that the coefficients of policy dummy variables are all positive and at a significant level of 1 or 5% all the time. It means that the impact of AAQS on green innovation of enterprises is not affected by the implementation of green credit and green finance reform and innovation pilot zone.

As for other policies except for green credit and green finance reform and innovation pilot zone, we cannot answer whether it has an impact on green innovation or not in this section. It has been listed as a limitation and will be overcome in future research.

### Heterogeneity analysis

According to some related literature, an enterprise's green innovation activities are influenced by the following heterogeneous factors, including the pollution degree of the industry to which the enterprise belongs (47, 57), the ownership attributes of the enterprise (8), the scale of the enterprise (47), the region where the enterprise locates (3, 31), and whether the listed company belongs to the patent-intensive industry (10). In the heterogeneity analysis, we classify the entire samples through five grouping dummy variables, including *Industry*_*it*_, *Owner*_*it*_, *Scale*_*it*_, *Region*_*it*_, *Intensive*_*it*_. Therefore, this study introduces the interaction term *D*_*it*_ × *Industry*_*it*_, *D*_*it*_ × *Owner*_*it*_, *D*_*it*_ × *Scale*_*it*_, *D*_*it*_ ×*Region*_*it*_,*D*_*it*_ × *Intensive*_*it*_ in Equation (1) to examine whether the impact of AAQS on green innovation of enterprises has the above five heterogeneous characteristics. Accordingly, we construct the triple-differences model as shown below, including Equations (3)–(7).


(3) $$\begin{array}{l} \text{Innovatio}n_{it}=\alpha +\beta_{1}D_{it}+\beta_{2}(D_{it\;}\times \;\text{Industr}y_{it})\\ \;+\;\gamma_{1}\text{contro}l_{it}+\mu_{i}+\lambda_{t}+\varepsilon_{it} \end{array}$$

(4) $$\begin{array}{l} \text{Innovatio}n_{it}=\alpha +\beta_{1}D_{it}+\beta_{2}(D_{it\;}\times \;\text{Owne}r_{it})\\ \;+\;\gamma_{2}\text{contro}l_{it}+\mu_{i}+\lambda_{t}+\varepsilon_{it} \end{array}$$

(5) $$\begin{array}{l} \text{Innovatio}n_{it}=\alpha +\beta_{1}D_{it}+\beta_{2}(D_{it\;}\times \;\text{Scal}e_{it})\\ \;+\;\gamma_{3}\text{contro}l_{it}+\mu_{i}+\lambda_{t}+\varepsilon_{it} \end{array}$$

(6) $$\begin{array}{l} \text{Innovatio}n_{it}=\alpha +\beta_{1}D_{it}+\beta_{2}(D_{it\;}\times \text{Regio}n_{it\;})\\ \;+\;\gamma_{4}\text{contro}l_{it}+\mu_{i}+\lambda_{t}+\varepsilon_{it} \end{array}$$

(7) $$\begin{array}{l} \text{Innovatio}n_{it}=\alpha +\beta_{1}D_{it}+\beta_{2}(D_{it\;}\times \;\text{Intensiv}e_{it})\\ \;+\;\gamma_{5}\text{contro}l_{it}+\mu_{i}+\lambda_{t}+\varepsilon_{it} \end{array}$$

In the heterogeneity analysis, there are five classifications for the entire sample. The first classification is in accordance with the pollution degree of the industry to which the enterprise belongs. We introduce *Industry*_*it*_ to classify the entire sample into heavy-polluting enterprises and non-heavily polluting enterprises. Regarding the identification of heavy-polluting enterprises, this study sifts out heavy-polluting enterprises by comparing “Guidelines for Industry Classification of Listed Companies” (http://www.csrc.gov.cn/csrc/) issued by the Securities Regulatory Commission with “List of Industry Classification Management of Listed Companies for Environmental Protection Verification” (http://www.gov.cn/gzdt/2008-07/07/) proposed by the Ministry of Environmental Protection (58). When the enterprise belongs to the heavy-polluting industry, the value of *Industry*_*it*_ is 1, otherwise the value of *Industry*_*it*_ is 0. The second classification is to classify the sample into state-owned enterprises and non-state-owned enterprises by introducing *Owner*_*it*_, according to different ownership attributes of the enterprises. If it is a state-owned enterprise, the value of *Owner*_*it*_ takes 1. If it is a non-state-owned enterprise, the value of *Owner*_*it*_ takes 0. The third classification is that we divide the sample into large-scale enterprises and small-scale enterprises, according to the 50th percentile of total assets of companies (8). The value of *Scale*_*it*_ is taken as 1 if it is a large-scale enterprise and taken as 0 if it is a small-scale enterprise. Furthermore, we classify the samples into eastern region, central and western regions in accordance with the region where the enterprise locates, which is the fourth classification. The central and western provinces in China are further merged according to the study of Yuan et al. (59). If enterprises are located in the eastern region, the value of *Region*_*it*_ is 1. For the enterprises located in the central and western regions, the value of *Region*_*it*_ is 0. The fifth classification is whether a listed company belongs to the patent-intensive industry. According to the “Catalogue of Patent-Intensive Industries” promulgated in 2016 (http://www.gov.cn/xinwen/2016-10/28/), we divided the entire sample into patent-intensive enterprises and non-patent-intensive enterprises (10). For patent-intensive enterprises, the value of *Intensive*_*it*_ takes 1, otherwise the value of *Intensive*_*it*_ takes 0.

This section uses Patent as the explained variable in the empirical analysis. Table 8 shows the results of heterogeneity analysis. Due to the limited space of the paper, we present the results of heterogeneity analysis when Inva and Uma are selected as the explained variable, respectively, in Supplementary Tables S2, S3 in the supplementary document, so as to make sure the study is comprehensive and rigorous. The findings of Supplementary Tables S2, S3 are consistent with the conclusions drawn from Table 8. We added the following interaction terms in columns (a)–(e), including *D*× *Industry, D*× *Owner, D*× *Scale, D*× *Region*, and *D*× *Intensive*, respectively. The results show that the coefficient of *D*× *Industry* is significantly positive, indicating that AAQS is more effective in promoting green innovation for companies in heavily polluting industries, compared with non-heavily polluting companies. The coefficient of *D*× *Owner* is 0.191, but not significant, which suggests that there is no significant difference between state-owned and non-state-owned enterprises in the promoting effect of AAQS on green innovation. The coefficient of *D*× *Scale* is significantly positive, which indicates that AAQS promotes green innovation more obviously in large-scale firms than in small-scale firms. Due to the constraints of capital, technology, and talent, environmental policies will lead to increased costs of pollution control, squeezing out R&D investment from small-scale enterprises. As a result, the level of green innovation will be reduced in small-scale enterprises. The coefficient of *D*× *Region* is 0.110 and passes the significance test at the 1% level. It indicates that compared with those in the central and western regions, AAQS has a more prominent impact on promoting green innovation activities of enterprises in the eastern region. Generally, enterprises in the central and western regions are relatively underdeveloped economically and have a weak capacity for independent innovation. The production of enterprises in the central and western regions mainly relies on imported technology or simple imitation, which causes a lot of waste of resources and environmental damage. Instead, eastern enterprises relocate industries with higher pollution degree to the central and western regions. Therefore, corporate technology innovation in the central and western regions has developed a path dependence on polluting technologies. After the implementation of AAQS, it is more difficult for companies in the central and western regions to transit to green technology innovation (3). The coefficient of *D*× *Intensive* is 0.141 and significant at the 1% level, indicating that compared with non-patent-intensive firms, AAQS significantly enhances the green innovation of patent-intensive enterprises. Patent-intensive industries have relatively more opportunities for technological transformation and technological upgrading flexibility. Environmental regulations will prompt enterprises to take the initiative to reduce production costs (10). Non-patent-intensive industries are vulnerable to technological homogenization and low-end constraints. The rising cost of environmental regulations will have a certain crowding-out effect on firms' R&D investment, resulting in lower green innovation capacity than patent-intensive industries (31).

**Table 8:** Results of heterogeneity analysis.

**Variables**	**Patent**				
	**(a)**	**(b)**	**(c)**	**(d)**	**(e)**
D	0.156**	0.062*	0.092***	0.110*	0.141***
	(0.104)	(0.082)	(0.081)	(0.099)	(0.082)
D × Industry	0.012**				
	(0.115)				
D × Owner		0.191			
		(0.108)			
D × Scale			0.140***		
			(0.108)		
D × Region				0.094*	
				(0.112)	
D × Intensive					0.071***
					(0.111)
Control variables	Control	Control	Control	Control	Control
Constant	1.363***	1.401***	1.323***	1.274***	1.252***
	(0.062)	(0.043)	(0.051)	(0.049)	(0.060)
Firm-fixed effect	Control	Control	Control	Control	Control
Year-fixed effect	Control	Control	Control	Control	Control
Observations	17,099	17,099	17,099	17,099	17,099
R-squared	0.724	0.702	0.787	0.790	0.762

The parentheses indicate the clustered standard errors at the prefecture-level firm level. ***, ** and * indicate significance at the 1%, 5% and 10% levels, respectively.

### Mechanism analysis

According to the theoretical analysis, it can be seen that environmental regulation affects green innovation of enterprises mainly through two impact mechanisms: the compliance cost effect and the innovation offset effect. Deng et al. (10) used firm production costs to represent the compliance cost effect and R&D investment to measure the innovation offset effect. Therefore, this study selects the operating cost rate (Cost) and R&D investment ratio (RD) as the mediating variables. We use the mediating effect model to verify the impact mechanism of AAQS on green innovation of enterprises. The definitions and descriptive statistics of the mediating variables are shown in Table 9. Referring to the studies of Zhang et al. (13) and Li and Liu (47), the mediating effect model is set as follows, including Equations (8)–(10).


(8) $$\begin{array}{lll} \text{Innovatio}n_{it} & = & cD_{it}+\delta_{1}\text{contro}l_{it}+{\mu_{i}+\lambda }_{t}+\varepsilon_{it} \end{array}$$

(9) $$\begin{array}{lll} M_{it} & = & aD_{it}+\delta_{2}\text{contro}l_{it}+{\mu_{i}+\lambda }_{t}+\varepsilon_{it} \end{array}$$

(10) $$\begin{array}{l} \text{Innovatio}n_{it}=c^{{\;}^{\prime }}D_{it}+bM_{it}+\delta_{3}\text{contro}l_{it}+\mu_{i}\\ \;+\lambda_{t}+\varepsilon_{it} \end{array}$$

**Table 9:** Descriptive statistics of the mediating variables.

**Statistic**	**Variable**	**Unit**	**Observations**	**Means**	**Standard**	**Min**	**Max**
	**definition**				**deviation**		
Cost	Operating costs/operating income	%	20,207	0.71067	0.21191	0	6.94275
RD	R&D investment/total corporate expenditure	%	20,254	2.92529	3.4703	0	20.8

where *M*_*it*_ is the mediating variable, including operating cost rate (Cost) and R&D investment ratio (RD), *c* denotes the total effect of AAQS on green innovation of firms, *c*′ represents the direct effect, and *ab* is the mediating effect.

In this section, Patent is used as the explained variable in the mechanism analysis. Due to the limited space of the paper, we present the results of mechanism analysis when Inva and Uma are selected as the explained variable in the supplementary document, including Supplementary Tables S4–S7, so as to make sure the study is comprehensive and rigorous. The findings of Supplementary Tables S4, S6 are consistent with the conclusions drawn from Table 10 in the manuscript, and the findings of Supplementary Tables S5, S7 are consistent with the conclusions drawn from Table 11 in the manuscript. The stepwise regression test of coefficients, Sobel test, and bootstrap test are used for mediating effects analysis (13, 60, 61). Table 10 shows the results of stepwise regression test of coefficients. We test the coefficient *c* based on Equation (8) in column (a). Columns (b) and (c) use operating cost ratio (Cost) and R&D investment ratio (RD) as mediating variables, respectively, to test the coefficient *a* in Equation (9). Columns (d) and (f) conduct the regression based on Equation (10) to obtain coefficients *c*′ and *b*, by using operating cost ratio (Cost) and R&D investment ratio (RD) as mediating variables, respectively. It can be seen that the coefficients *a*, *b*, and *c* are all significant, so the mediation effect is established (61). The results of columns (b) and (d) demonstrate the mechanism path of compliance cost effect. The coefficient in column (b) is 0.012 and significant at the 10% level, indicating that the implementation of AAQS leads to an average increase of 0.012 in the production costs of firms. Environmental regulations push companies investing money in fighting pollution, causing an increase in cost. Furthermore, the coefficient in column (d) is −0.076, which is significant at the 10% level, indicating that the increase in production cost of firms reduces the enterprises' green patent applications. Therefore, we conclude the mechanism path of compliance cost effect: the implementation of AAQS enhances the production costs of firms and further decreases the green innovation of enterprises, which supports hypothesis 2. From the results in columns (c) and (e), the mechanism path of the innovation offset effect can be derived. The coefficient in column (c) is 0.655 and passed the 1% significance test, indicating that the implementation of AAQS results in an average increase of 0.655 in corporate R&D investment. Moreover, the coefficient in column (e) is 0.021, which is significant at the 5% level, indicating that the increase in corporate R&D investment enhances the number of green patent applications. Hence, we conclude the mechanism path of innovation offset effect: the implementation of AAQS enhances firms' R&D investment, thus improving the green innovation of enterprises, which supports hypothesis 3.

**Table 10:** Results of stepwise regression test of coefficients.

**Variables**	**Equation (8)**	**Equation (9)**		**Equation (10)**	
	**Patent**	**Cost**	**RD**	**Patent**	**Patent**
	**(a)**	**(b)**	**(c)**	**(d)**	**(e)**
D	0.165**	0.012*	0.655***	0.153**	0.113*
	(0.069)	(0.011)	(0.211)	(0.033)	(0.092)
Cost				−0.076*	
				(0.043)	
RD					0.021**
					(0.010)
Control variables	Control	Control	Control	Control	Control
Constant	1.164***	0.700***	1.934***	1.219***	1.471***
	(0.109)	(0.017)	(0.623)	(0.117)	(0.181)
Firm-fixed effect	Control	Control	Control	Control	Control
Year-fixed effect	Control	Control	Control	Control	Control
Observations	17,099	17,099	17,099	17,099	17,099
R-squared	0.734	0.617	0.662	0.726	0.739

The parentheses indicate the clustered standard errors at the prefecture-level firm level. ***, **, and * indicate significance at the 1, 5, and 10% levels, respectively.

**Table 11:** Results of Sobel test and bootstrap test.

**Sobel test**	**Compliance cost effect** **(Mediating variable: Cost)**	**Innovation offset effect** **(Mediating variable: RD)**
P-value	0.02595	0.004256
Proportion of mediating effects	5.48431%	15.43527%
Control variables	Control	Control
**Bootstrap test**	**Compliance cost effect** **(Mediating variable: Cost)**	**Innovation offset effect** **(Mediating variable: RD)**
Confidence interval	[−0.09712, −0.05318]	[0.01478, 0.20744]
Control variables	Control	Control

In this section, the Sobel test and bootstrap test are used to further verify the compliance cost effect and the innovation compensation effect. Sobel test shows the *p-*value and the proportion of mediating effect. The bootstrap test presents the confidence interval with 95% confidence level obtained by randomly sampling 1000 times estimation. The results of Sobel test and bootstrap test are presented in Table 11. It can be seen that the *p-*value of the compliance cost effect is 0.02595, which is < 0.05, and the confidence interval does not contain 0. Therefore, it is further verified that the mediating effect of corporate production cost on green innovation holds; that is, the compliance cost effect is valid. Furthermore, the *p-*value of the innovation offset effect is 0.004256, which is also < 0.05, and the confidence interval does not contain 0. Therefore, it is further suggested that the mediating effect of corporate R&D investment on green innovation holds; that is, the innovation compensation effect is established. In addition, the study calculates the proportion of the mediating effect of the two impact mechanisms: the percentage of compliance cost effect is 5.48431% while the percentage of innovation offset effect is 15.43527%. It indicates that the innovation offset effect of AAQS is greater than the compliance cost effect, which confirms hypothesis 4. Therefore, the positive impact of the innovation offset effect exceeds the negative impact caused by the compliance cost effect, which indicates that the total effect of AAQS on the green innovation of enterprises is positive. This is the internal mechanism that explains why AAQS improves the green innovation of enterprises.

## Discussion

It is practical and critical for enterprises to promote their green innovation level through production cost control and R&D investment input. Meanwhile, governments should formulate and implement appropriate environmental regulation to support firms to put more efforts into green innovation, thus contributing to environmental protection and public health. This study makes a significant contribution to exploring the policy effects of environmental regulation on firms' green innovation and putting forward the implications for environmental protection and public health. Based on the panel data of Chinese A-share listed companies from 2008 to 2020, this paper analyzes the policy effect of Ambient Air Quality Standard implemented in China in 2012 on green innovation of enterprises, heterogeneity characteristics, and impact mechanisms. Taking the Ambient Air Quality Standard as a quasi-natural experiment, this study successively adopts the DID model, PSM-DID, triple-differences model, and mediating effect methods in the empirical analysis.

The DID model is carried out by using three metrics of the green innovation, including the number of green patent applications, the number of green invention patent applications, and the number of green utility model patent applications, which is similar to the studies of Wu et al. (49) and Ma et al. (52). We find that AAQS significantly enhances the green innovation of firms, which verifies our hypothesis 1. It is consistent with the conclusions of many existing literatures (9–11). In terms of heterogeneity analysis, this study comprehensively examines the differences in the effects of AAQS on corporate green innovation in the following five aspects: the pollution degree of the industry to which the enterprise belongs, the ownership attributes of the enterprise, the scale of the enterprise, the region where the enterprise locates, and whether the enterprise belongs to the patent-intensive industry. Although some studies also verified one or more of these heterogeneous characteristics separately, they fail to combine the above heterogeneous characteristics together simultaneously for heterogeneity analysis (8, 10, 47).

This study examines two mechanism paths by using the mediating effect: one is the compliance cost effect, and the other is the innovation offset effect, where AAQS enhances green innovation by virtue of firms' production costs and R&D investment (3, 31), which supports our hypothesis 2 and hypothesis 3. We have proved that the innovation compensation effect of AAQS is greater than the compliance cost effect, which confirms our hypothesis 4. It indicates that the total effect of AAQS on the green innovation of enterprises is positive. However, the conclusion is contrary to the study of Wang et al. (2), which found that the compliance cost effect of environmental regulation is greater than the innovation compensation effect, leading to a negative total effect. Some studies conduct quite a different mechanism analysis (2, 10), which adopted moderating effect in the mechanism analysis to test the effect of environmental enforcement, public scrutiny, and media scrutiny driven by AAQS on green innovation.

Overall, promoting corporate green innovation should be long-term strategic goal, which requires the support and supervision of governments' environmental regulation. The corporate reputation should also not be too disturbed by increased production cost, declined R&D investment, regional differences, and industrial differences. Otherwise, enterprises' green innovation will be damaged and thus not be conducive to environmental protection and public health. The findings of the study provide implications for governments to formulate environmental policies that promote green technology innovation of firms, mitigate environmental pollution, and improve public health.

## Conclusions and implications

### Main findings

The research on the impact and intrinsic mechanism of AAQS on corporate green innovation has significant importance for environmental protection and public health. The implementation of AAQS can significantly affect corporate green innovation. However, a dilemma exists; that is, the compliance cost effect is negative while the innovation offset effect is positive. Whether innovation offset effect is greater than the compliance cost effect and how the implementation of AAQS affects green innovation by virtue of the two mechanism paths are becoming increasingly critical for academic research and policy formulation to promote environmental protection and public health. The main findings are summarized as follows.

First, the implementation of AAQS significantly improves the green innovation of enterprises. After a series of robustness tests including parallel trend test, placebo test, replacement of the explained variables, and PSM-DID estimation, the positive effect remains valid.

Second, the policy effect of AAQS on firms' green innovation has heterogeneous characteristics. We find that AAQS is more effective in promoting green innovation of firms in heavily polluting industries than those in non-heavily polluting industries. There is no significant difference in the policy effect of AAQS between state-owned and non-state-owned enterprises. AAQS has a more prominent impact in promoting green innovation for large-scale enterprises than for small-scale enterprises. Compared with enterprises in the central and western regions, the effect of AAQS on green innovation is more pronounced than those in the eastern region. Besides, AAQS has more significant effect on green innovation in patent-intensive companies than in non-patent-intensive firms.

Third, the mechanism analysis shows that environmental regulation influences the green innovation of enterprises mainly through two mechanisms: the compliance cost effect and the innovation offset effect. On the one hand, the implementation of AAQS leads to an increase in the production costs of enterprises, thus inhibiting green innovation activities of enterprises. On the other hand, under the policy guidance of AAQS, enterprises increase their R&D investment in green technology, thus enhancing their green innovation. Moreover, the positive impact of the innovation offset effect is greater than the negative impact caused by the compliance cost effect, which indicates that the total effect of AAQS on the green innovation of enterprises is positive.

### Theoretical implications

This study serves as a bridge to environmental regulation, green innovation, environmental protection, and public health and contributes to the existing literature as follows.

First, the research on AAQS and green innovation enriches the existing studies of the impact of environmental regulation on green innovation of enterprises. Taking the implementation of AAQS as a quasi-natural experiment, this study introduces the compliance cost effect and the innovation offset effect (3) and investigates how the implementation of AAQS influences green innovation by DID model, which can be considered an innovative approach to policy assessment. The combination of theoretical analysis and empirical analysis provides a great research method for the future of scientific literature.

Second, a series of empirical analysis in detail depicts the impact and intrinsic mechanism of AAQS on green innovation of enterprises, which include heterogeneity analysis, mechanism analysis, PSM-DID, and excluding the impact of other policies. The existing literature proved that AAQS can enhance corporate green innovation. However, the intrinsic mechanism and heterogeneous characteristics of AAQS on green innovation need to be further investigated. Triple-differences model, mediating effect analysis, and PSM-DID estimation provide a new perspective to conduct thorough empirical research.

Third, we extend and enrich the compliance cost effect and the innovation offset effect and apply them to the research on the intrinsic mechanism of AAQS affecting green innovation of enterprises. These results verify the theoretical assumption and demonstrate that government should encourage firms to contribute more to environmental protection and public health from the production cost and R&D investment perspective.

### Policy implications

Our findings not only enrich the existing studies of environmental regulation and green innovation of enterprises, but also provide implications for policymakers in China and other developing countries on how to promote green innovation of enterprises, for the improvement of environment and public health. Based on the empirical conclusions of our research, we derive the following policy implications for ecological environment protection and public health improvement.

First, more attention should be paid to the investment in green technology innovation when governments formulate environmental policies. From the perspective of innovation offset effect, policymakers should increase support for enterprises' R&D investment, by providing them with credit support, financial tax preferences, and administrative rewards (62). The essential of these measures is to exert the innovation compensation effect in promoting the green innovation of enterprises. Furthermore, governments should shorten the time of green patent approval and simplify the green patent approval process, which contributes to increase the willingness of enterprises to carry out green transformation and green innovation, thus leading to less environmental pollution and a good public health state.

Second, more efforts should be devoted to the reduction of firms' cost of pollution control. In response to the environmental regulation promulgated by governments, enterprises will purchase emission-reductive equipment and relocate heavy-polluting sectors to cope with the regulated emission standard during the production process (63). We conclude from the mechanism analysis that environmental regulation leads to an increase in production costs of enterprises, thus inhibiting green innovation activities of enterprises. Hence, governments should implement environmental subsidies for enterprises to reduce the cost of equipment purchase and emission control. By reducing the cost of firms' pollution control, policymakers can prevent enterprises from increasing production costs caused by pollution control, thus inhibiting green technology innovation. The core of the above measures is to mitigate the negative impact of compliance cost effect. It is helpful to increase the quality level of public health.

Third, policymakers need to consider the heterogeneous characteristics of enterprises when formulating environmental policies (64). Authorities should implement differentiated environmental regulation according to the pollution degree of the industry, the ownership attributes of the enterprise, the scale of the enterprise, the region where the enterprise locates, and whether the enterprise belongs to the patent-intensive industry. More emphasis should be placed on heavy-polluting enterprises, large-scale enterprises, enterprises in economically developed regions, and patent-intensive enterprises. Governments should provide policy preferences and subsidies to support green technology innovation for the above enterprises, which provide green products to reduce environmental pollution. It is beneficial for public health soundness.

### Limitations and future work

There are several limitations and future directions to fulfill in this study. First, different types of environmental regulations could have distinct intensity of effects on green innovation of enterprises. We merely study the policy implications and mechanisms of AAQS, a type of command-and-control environmental regulation, but fail to consider market-based incentive and public participation regulations. Second, we have excluded the impact of the two policies including green credit and green finance reform and innovation pilot zone (GFRIPZ) on green innovation. However, as for other policies except for green credit and GFRIPZ, we cannot answer whether it has an impact on green innovation or not. Third, we fail to confirm these green innovations are related to air quality control; that is, we cannot distinguish which is green innovation application specifically toward air quality standard and which is not. These limitations will be overcome in future research. Our future work will focus on the policy effects of a wide range of environmental regulations on green innovation and find out the approach to solve the above current limitations.

## Data availability statement

The datasets presented in this study can be found in online repositories. The names of the repository/repositories and accession number(s) can be found in the article/Supplementary material.

## Author contributions

Z-fZ: visualization, supervision, and validation. H-dX: conceptualization, methodology, writing—original draft, and writing—review and editing. S-sS: data curation, software, and visualization. H-yD: conceptualization and writing—review and editing. Y-qL: methodology and data curation. All authors listed have made a substantial, direct, and intellectual contribution to the work and approved it for publication.

## Funding

This research was supported by the Cooperation and Innovation International Project Fund (2021-HSZ029) and the Qingdao Social Science Planning Project (QDSKL2001410).

## Conflict of interest

The authors declare that the research was conducted in the absence of any commercial or financial relationships that could be construed as a potential conflict of interest.

## Publisher's note

All claims expressed in this article are solely those of the authors and do not necessarily represent those of their affiliated organizations, or those of the publisher, the editors and the reviewers. Any product that may be evaluated in this article, or claim that may be made by its manufacturer, is not guaranteed or endorsed by the publisher.
