# Supplementary material for: Does ambient air quality standard contribute to green innovation of enterprises in China? Implications for environmental protection and public health
Source: Front Public Health. 2022 Nov 10;10:997864. doi: 10.3389/fpubh.2022.997864 (PMC9687091; doi:10.3389/fpubh.2022.997864)
Supplement: Supplementary file 1 [file Data_Sheet_1.docx]

Does Ambient Air Quality Standard Contribute to Green Innovation of Enterprises in China? Implications for Environmental Protection and Public Health (Supplementary Material)

# Supplementary Data

We have uploaded data file separately on submission.

# Supplementary Figures and Tables

**2.1 Supplementary Figures**

We have placed the figures (labeled in order of appearance in the manuscript) in a zip file and upload them as 'Supplementary Material Presentation.'

**2.2 Supplementary Tables**

The supplementary tables here include Table S1, Table S2, Table S3, Table S4, Table S5, Table S6 and Table S7, which are associated with the manuscript.

**Table S1 |** List of 74 pilot cities in the “Ambient Air Quality Standard Phase I Monitoring Implementation Plan”.

| **No.** | **City** | **AQI** | **No.** | **City** | **AQI** | **No.** | **City** | **AQI** |
| --- | --- | --- | --- | --- | --- | --- | --- | --- |
| 1 | Shijiazhuang | 92.2 | 26 | Changchun | 39.2 | 51 | Jinhua | 30.4 |
| 2 | Xingtai | 89.0 | 27 | Huhehaote | 38.4 | 52 | Shanghai | 29.5 |
| 3 | Baoding | 78.7 | 28 | Yangzhou | 37.8 | 53 | Taizhou | 29.5 |
| 4 | Xian | 77.8 | 29 | Zhangjiakou | 37.7 | 54 | Guangzhou | 29.4 |
| 5 | Handan | 75.8 | 30 | Chongqing | 37.5 | 55 | Nantong | 29.3 |
| 6 | Wulumuqi | 73.9 | 31 | Changzhou | 37.4 | 56 | Wenzhou | 29.0 |
| 7 | Jinan | 71.8 | 32 | Lianyungang | 37.4 | 57 | Foshan | 28.6 |
| 8 | Hengshui | 65.8 | 33 | Huaian | 37.1 | 58 | Kunming | 28.5 |
| 9 | Tangshan | 65.4 | 34 | Nanchang | 36.9 | 59 | Ningbo | 27.7 |
| 10 | Zhengzhou | 62.1 | 35 | Suqian | 36.8 | 60 | Xiamen | 27.2 |
| 11 | Chengdu | 56.5 | 36 | Taizhou | 36.7 | 61 | Zhongshan | 27.1 |
| 12 | Langfang | 54.9 | 37 | Yancheng | 34.5 | 62 | Dongguan | 26.7 |
| 13 | Taiyuan | 52.2 | 38 | Wuxi | 34.3 | 63 | Quzhou | 26.0 |
| 14 | Cangzhou | 51.8 | 39 | Hefei | 34.0 | 64 | Shenzhen | 24.8 |
| 15 | Tianjin | 51.2 | 40 | Changsha | 34.0 | 65 | Guiyang | 24.5 |
| 16 | Qinhuangdao | 48.7 | 41 | Dalian | 33.9 | 66 | Zhuhai | 24.4 |
| 17 | Xuzhou | 48.6 | 42 | Huzhou | 33.6 | 67 | Jiangmen | 24.2 |
| 18 | Beijing | 47.2 | 43 | Rongqing | 33.2 | 68 | Nanning | 23.3 |
| 19 | Shenyang | 4.58 | 44 | Hangzhou | 3.21 | 69 | Huizhou | 2.28 |
| 20 | Wuhan | 4.30 | 45 | Chengde | 3.20 | 70 | Tianshui | 2.28 |
| 21 | Xining | 4.29 | 46 | Lanzhou | 3.17 | 71 | Lasa | 2.17 |
| 22 | Harbin | 4.16 | 47 | Zhenjiang | 3.13 | 72 | Fuzhou | 2.13 |
| 23 | Yinchuan | 4.06 | 48 | Jiaxing | 3.13 | 73 | Zhoushan | 1.85 |
| 24 | Qingdao | 4.02 | 49 | Shaoxing | 3.10 | 74 | Haikou | 1.62 |
| 25 | Nanjing | 3.96 | 50 | Suzhou | 3.08 |  |  |  |

*Note: AQI indicates air quality index*

**Table S2 |** Results of heterogeneity analysis: Inva is selected as the explained variable.

| **Variables** | **Inva** | | | | |
| --- | --- | --- | --- | --- | --- |
|  | **(a)** | **(b)** | **(c)** | **(d)** | **(e)** |
| D | 0.032* | 0.029*** | 0.035** | 0.026* | 0.072* |
|  | (0.043) | (0.044) | (0.059) | (0.050) | (0.042) |
| D× Industry | 0.146*** |  |  |  |  |
|  | (0.051) |  |  |  |  |
| D× Owner |  | 0.097 |  |  |  |
|  |  | (0.057) |  |  |  |
| D× Scale |  |  | 0.088** |  |  |
|  |  |  | (0.058) |  |  |
| D× Region |  |  |  | 0.094* |  |
|  |  |  |  | (0.059) |  |
| D× Intensive |  |  |  |  | 0.026** |
|  |  |  |  |  | (0.064) |
| Control variables | Control | Control | Control | Control | Control |
| Firm-fixed effect | Control | Control | Control | Control | Control |
| Year-fixed  effect | Control | Control | Control | Control | Control |
| Observations | 17099 | 17099 | 17099 | 17099 | 17099 |
| R-squared | 0.738 | 0.762 | 0.750 | 0.609 | 0.686 |

*Note: The parentheses indicate the clustered standard errors at the prefecture-level firm level. ***, ** and * indicate significance at the 1%, 5% and 10% levels, respectively.*

Table S2 presents the results of heterogeneity analysis when we use Inva as the explained variable. The findings are consistent with the conclusions drawn from Table 8 in the manuscript.

**Table S3 |** Results of heterogeneity analysis: Uma is selected as the explained variable.

| **Variables** | **Uma** | | | | |
| --- | --- | --- | --- | --- | --- |
|  | **(a)** | **(b)** | **(c)** | **(d)** | **(e)** |
| D | 0.171** | 0.086* | 0.098*** | 0.125* | 0.150* |
|  | (0.101) | (0.080) | (0.078) | (0.097) | (0.080) |
| D× Industry | 0.023* |  |  |  |  |
|  | (0.112) |  |  |  |  |
| D× Owner |  | 0.164 |  |  |  |
|  |  | (0.106) |  |  |  |
| D× Scale |  |  | 0.143** |  |  |
|  |  |  | (0.106) |  |  |
| D× Region |  |  |  | 0.084* |  |
|  |  |  |  | (0.110) |  |
| D× Intensive |  |  |  |  | 0.070*** |
|  |  |  |  |  | (0.108) |
| Control variables | Control | Control | Control | Control | Control |
| Firm-fixed effect | Control | Control | Control | Control | Control |
| Year-fixed  effect | Control | Control | Control | Control | Control |
| Observations | 17099 | 17099 | 17099 | 17099 | 17099 |
| R-squared | 0.690 | 0.714 | 0.706 | 0.726 | 0.775 |

*Note: The parentheses indicate the clustered standard errors at the prefecture-level firm level. ***, ** and * indicate significance at the 1%, 5% and 10% levels, respectively.*

Table S3 presents the results of heterogeneity analysis when we use Uma as the explained variable. The findings are consistent with the conclusions drawn from Table 8 in the manuscript.

**Table S4 |** Results of stepwise regression test for coefficients: Inva is selected as the explained variable**.**

| **Variables** | **Equation（8）** | **Equation（9）** | | **Equation（10）** | |
| --- | --- | --- | --- | --- | --- |
|  | **Inva** | **Cost** | **RD** | **Inva** | **Inva** |
|  | **(a)** | **(b)** | **(c)** | **(d)** | **(e)** |
| D | 0.081** | 0.012* | 0.655*** | 0.072** | 0.027* |
|  | (0.038) | (0.011) | (0.211) | (0.018) | (0.053) |
| Cost |  |  |  | -0.025* |  |
|  |  |  |  | (0.026) |  |
| RD |  |  |  |  | 0.016*** |
|  |  |  |  |  | (0.005) |
| Control variables | Control | Control | Control | Control | Control |
| Constant | 0.260*** | 0.700*** | 1.934*** | 0.243*** | 0.352*** |
|  | (0.067) | (0.017) | (0.623) | (0.070) | (0.102) |
| Firm-fixed effect | Control | Control | Control | Control | Control |
| Year-fixed effect | Control | Control | Control | Control | Control |
| Observations | 17099 | 17099 | 17099 | 17099 | 17099 |
| R-squared | 0.754 | 0.617 | 0.662 | 0.717 | 0.767 |

*Note: The parentheses indicate the clustered standard errors at the prefecture-level firm level. ***, ** and * indicate significance at the 1%, 5% and 10% levels, respectively.*

Table S4 presents the results of mechanism analysis when we use Inva as the explained variable. The findings are consistent with the conclusions drawn from Table 10 in the manuscript.

**Table S5 |** Results of sobel test and bootstrap test: Inva is selected as the explained variable**.**

| **Sobel test** | **Compliance cost effect**  **(Mediating variable：Cost)** | **Innovation offset effect**  **(Mediating variable：RD)** |
| --- | --- | --- |
| P-value | 0.0360989 | 0.0043463 |
| Proportion of Mediating effects | 5.83604% | 20.63444% |
| Control variables | Control | Control |
| **Bootstrap test** | **Compliance cost effect**  **(Mediating variable：Cost)** | **Innovation offset effect**  **(Mediating variable：RD)** |
| Confidence interval | [-0.028851, -0.008055] | [0.0315997, 0.1453972] |
| Control variables | Control | Control |

The findings in Table S5 are consistent with those drawn from Table 11 in the manuscript.

**Table S6 |** Results of stepwise regression test for coefficients: Uma is selected as the explained variable**.**

| **Variables** | **Equation（8）** | **Equation（9）** | | **Equation（10）** | |
| --- | --- | --- | --- | --- | --- |
|  | **Uma** | **Cost** | **RD** | **Uma** | **Uma** |
|  | **(a)** | **(b)** | **(c)** | **(d)** | **(e)** |
| D | 0.173** | 0.012* | 0.655*** | 0.132** | 0.106*** |
|  | (0.067) | (0.011) | (0.211) | (0.053) | (0.024) |
| Cost |  |  |  | -0.051* |  |
|  |  |  |  | (0.038) |  |
| RD |  |  |  |  | 0.016*** |
|  |  |  |  |  | (0.005) |
| Control variables | Control | Control | Control | Control | Control |
| Constant | 1.114*** | 0.700*** | 1.934*** | 1.827*** | 0.168*** |
|  | (0.102) | (0.017) | (0.623) | (0.251) | (0.034) |
| Firm-fixed effect | Control | Control | Control | Control | Control |
| Year-fixed effect | Control | Control | Control | Control | Control |
| Observations | 17099 | 17099 | 17099 | 17099 | 17099 |
| R-squared | 0.745 | 0.617 | 0.662 | 0.733 | 0.702 |

*Note: The parentheses indicate the clustered standard errors at the prefecture-level firm level. ***, ** and * indicate significance at the 1%, 5% and 10% levels, respectively.*

Table S6 presents the results of mechanism analysis when we use Uma as the explained variable. The findings are consistent with the conclusions drawn from Table 10 in the manuscript.

**Table S7 |** Results of sobel test and bootstrap test: Uma is selected as the explained variable**.**

| **Sobel test** | **Compliance cost effect**  **(Mediating variable：Cost)** | **Innovation offset effect**  **(Mediating variable：RD)** |
| --- | --- | --- |
| P-value | 0.0111913 | 0.0042907 |
| Proportion of Mediating effects | 6.65516% | 16.30232% |
| Control variables | Control | Control |
| **Bootstrap test** | **Compliance cost effect**  **(Mediating variable：Cost)** | **Innovation offset effect**  **(Mediating variable：RD)** |
| Confidence interval | [-0.05736, -0.01613] | [0.02876, 0.19295] |
| Control variables | Control | Control |

The findings in Table S7 are consistent with those drawn from Table 11 in the manuscript.
